# What Is Known About Eating Disorders and Disordered Eating in Individuals With Physical Disabilities? A Narrative Review

**DOI:** 10.1111/cch.70184

**Published:** 2025-11-14

**Authors:** Revi Bonder, Meaghan Walker, Alene Toulany, Amy C. McPherson

**Affiliations:** ^1^ Bloorview Research Institute, Holland Bloorview Kids Rehabilitation Hospital Toronto Ontario Canada; ^2^ Division of Adolescent Medicine, Sick Kids Hospital Toronto Ontario Canada; ^3^ Department of Paediatrics University of Toronto Toronto Ontario Canada

**Keywords:** body image, disordered eating, eating disorders, physical disabilities

## Abstract

Individuals with physical disabilities may be at a high risk for developing eating disorders/disordered eating (ED/DE) and body image concerns yet are often excluded from research in this field. This has created a critical gap in our understanding of eating patterns and body image concerns among individuals with disabilities, which may impact physical and mental health outcomes long term. This narrative review explores existing literature on ED/DE and body image in individuals with physical disabilities and identifies unique factors that may increase risk. Time‐sensitive, developmentally appropriate and specialized treatment options are needed to support this population.

## Summary

- Eating disorders (ED) have the highest mortality rate among psychiatric conditions and disordered eating (DE) affects millions, yet little is known about their impact on individuals with physical disabilities.
- Unique risk factors—like body image issues, feeding challenges, mobility concerns, social stigma and ableism—can make people with physical disabilities more vulnerable to ED/DE.
- ED/DE in this population are often overlooked in clinical settings, highlighting the need for specialized training.
- Research on ED/DE among people with physical disabilities is limited, especially for specific disability subgroups. More studies are needed on prevalence, presentation and tailored interventions.

## 1 Introduction

Eating disorders (EDs) have the highest mortality rate of any psychiatric disorders (Miskovic‐Wheatley et al. 2023). While the DSM‐5 outlines criteria for what constitutes a diagnosable ED, (Canadian Eating Disorder Alliance 2019), disordered eating (DE) is a broader concept that includes a large spectrum of eating‐related behaviours. These may include restricted food intake, weight cycling (i.e., regularly losing and gaining weight), caloric restriction, deliberately skipping meals, overeating/binging and compensatory behaviours such as purging (Brownell and Walsh 2017; Bonder et al. 2025). While these behaviours are part of a formal ED diagnostic criteria, in the context of DE, these behaviours can occur at subclinical levels (e.g., less frequently, lower intensity) and therefore may not meet the threshold for a clinical diagnosis. DE is multifactorial in nature (Pereira and Alvarenga 2007) with the aetiology impacted by numerous biopsychosocial factors (Canadian Eating Disorder Alliance 2019; Brownell and Walsh 2017; Bonder et al. 2025).

Understandably, the prevalence of DE is challenging to determine, as those engaging in DE often do not seek professional help, and distress may be subjective (Canadian Eating Disorder Alliance 2019; Volpe et al. 2016). Individuals engaged in DE may not fully recognize or disclose the extent of their behaviours, particularly because such behaviours often start in adolescence (Canadian Eating Disorder Alliance 2019; Volpe et al. 2016). Nonetheless, it is estimated that DE affects millions of Canadians each year (McVey et al. 2005).

ED/DE are both detrimental to physical and psychosocial health and wellbeing (Bonder et al. 2025; American Psychiatric Association 2013; National Eating Disorders Association, n.d.; Silber et al. 1999). Physical consequences can include malnutrition, growth failure, fatigue, anaemia, amenorrhea and hair loss (American Psychiatric Association 2013; National Eating Disorders Association, n.d.; Silber et al. 1999). Frequently, there are mental health comorbidities, such as anxiety, depression, obsessive compulsive disorder and social phobia (American Psychiatric Association 2013; National Eating Disorders Association, n.d.; Silber et al. 1999). Individuals with ED/DE also often have poorer body image—the self‐perception and evaluation of one's body. Emerging work (Steinhoff et al. 2025) discusses the intersectionality between EDs/DE and disabilities, suggesting that the two conditions are also mutually reinforcing. For example, medical complications caused by EDs, such as osteoporosis, can exacerbate existing disability‐related impairments or symptoms. Alternatively, disability‐related stigma can increase feelings of body dissatisfaction and contribute to vulnerability to EDs/DE.

While there is a growing recognition of the prevalence and presentation of ED/DE in the general population (Sim et al. 2010), there has been much less exploration among historically marginalized groups, such as Indigenous and People of Colour (BIPOC) and the 2SLGBTQIA + community (Bardone‐Cone et al. 2017; Parker and Harriger 2020). Extremely little attention has been given to how ED/DE impacts individuals with disabilities, despite 27% of Canadians (~8 million people) and 16% (~1.8 billion people) of people worldwide reporting having a disability (Statistics Canada 2022). This has created a significant gap in our understanding of both the aetiology and treatment of both ED and DE in people with disabilities, leaving potentially serious physical and mental health risks unaddressed.

## 2 The Current Review

The main objective of this narrative review is to identify and describe the existing literature on ED/DE in individuals with physical disabilities (IWPD). While ED and DE are not interchangeable terms, during a preliminary search, there was insufficient literature to focus upon each one separately. Furthermore, specific definitions were not provided in every paper that discussed potentially problematic eating behaviours. We chose to explore this phenomenon across the lifespan, because while ED/DE often start in young people, they are often experienced throughout a person's life (Breton et al. 2022). Further, we chose to specifically focus on physical disabilities given that EDs in neurodevelopmental and/or intellectual disabilities have been much more widely studied (Schröder et al. 2022; Gravestock 2000). There are many definitions of physical disability. For the purpose of this review, we draw upon the World Health Organization's International Classification of Functioning, Disability and Health (ICF) model, which defines physical disability as a bodily impairment that interferes with a person's physical function (World Health Organization 2011). Specifically, we focused on mobility‐related and neuromuscular conditions such as spina bifida, cerebral palsy (CP) and Duchenne muscular dystrophy. We excluded conditions primarily related to sensory function (e.g., vision or hearing loss), intellectual or developmental disabilities and chronic health conditions not primarily associated with physical impairment (e.g., asthma, cystic fibrosis). Our aim was to explore existing research evidence to identify the factors that contribute to ED/DE in IWPD and the implications for treatment.

### 2.1 Narrative Review Methodology

Narrative reviews are often employed when seeking an initial understanding of a body of literature. They offer a helpful summary of relevant and current knowledge on a specific topic, while simultaneously highlighting new perspectives and unveiling new questions yet to be explored by the literature (Ferrari 2015; Greenhalgh et al. 2011; Rother 2007). Because of the limited and methodologically diverse nature of the literature on ED/DE in IWPD identified in the preliminary search, a narrative review was deemed an appropriate methodological approach to report on the state of literature to date (Rother 2007).

Articles selected for inclusion in the narrative review met the following inclusion criteria: (1) available in English; (2) published during or after 2000 (for comprehensive coverage) and (3) focused on ED or DE in IWPD. Articles were excluded if they primarily focused upon the following: (1) participants with chronic health conditions (e.g., asthma, sickle cell disease) or sensory disabilities (e.g., visual or hearing impairment); (2) intellectual disabilities or autism spectrum disorder and (3) other feeding and EDs, such as avoidant/restrictive food intake disorder (ARFID). We chose to exclude individuals with chronic health conditions, due to the implications their health condition, and subsequent medications and/or treatment, can have on eating behaviours and food tolerance. We chose to exclude articles about feeding disorders such as ARFID, given that the causes and mechanisms are considered to be different from other EDs. For example, food avoidance in ARFID is typically not motivated by body shape and weight concerns as with anorexia nervosa (Stern et al. 2024).

To search the literature, key terms related to disability, EDs and DEs were searched. Databases including PsycInfo, Scopus and Web of Science were searched, with Scopus and Web of Science including health and social sciences databases like MEDLINE, Embase and ProQuest. Google Scholar was also searched for additional literature, including dissertations, preprints and technical reports. The reference lists of included articles were reviewed to find further relevant studies. Articles meeting inclusion criteria were summarized by two research team members (MW and RB). Then, through collaborative discussions with the broader research team, common patterns were identified and grouped into overarching themes and subthemes. Final themes were selected based on their ability to meaningfully capture key insights across the literature. The research team represented expertise in health psychology, clinical psychology, occupational therapy, paediatric medicine, childhood disability, mental health and weight stigma.

## 3 Findings

Our search identified 29 articles that met our criteria. Two key themes arose from our findings: (1) disability‐specific characteristics contribute to food restriction and (2) ableism impacting the development of ED/DE. For each theme, two subthemes were identified. For the first theme, subthemes were: nonweight‐related influences on eating patterns and other motivations for IWPD to reduce their weight. For the second theme, subthemes were: the connection between disability and body image; and weight loss as a mechanism to ameliorate ableism. Additional literature was then used to contextualize our findings.

### 3.1 Disability‐Specific Characteristics Contribute to Food Restriction

#### 3.1.1 Nonweight‐Related Influences on Eating Patterns

The literature discussed a multitude of disability‐specific characteristics that can increase the risk of ED/DE in IWPD by influencing eating behaviours. For example, articles described that some IWPD may restrict food and fluids to avoid bowel and urinary disturbances, due to incontinence and/or increased difficulty with independent toileting (Hershenson 2016). A study with youth attending a spina bifida outpatient clinic found that bowel and urinary ‘accidents’ were a frequent worry for participants and they restricted their food and fluid intake during the day to avoid having an incontinence episode during school (McPpherson et al. 2013). Similarly, a case study of a 33‐year‐old woman with CP reported that she was embarrassed about her incontinence overnight, because she was reliant on nursing staff for toileting. In an attempt to avoid embarrassment, she reported not consuming any food or beverages in the latter half of the day (Webb et al. 2009).

Additionally, food consumption can be impacted by both physiological factors and factors related to physical function and coordination. Brain malformations associated with certain physical disabilities (e.g., Chiari malformation) can impact taste and texture preference, which can affect eating patterns and potentially exclude nutritious food that is more fibrous (Hershenson 2016).

Some IWPD also experience decreased fine and gross motor skills, which can make the act of feeding oneself more difficult, resulting in decreased intake. One study discussed an adult with a physical disability and coordination challenges who did not eat in public because she felt ‘messy’ and ‘disgusted’ with herself (Webb et al. 2009). IWPD may also be dependent on caregivers to assist with feeding (Dillenburger and Mckerr 2014; Walker et al. 2019). The increased dependence for feeding has been found to increase the likelihood of developing an ED (Cicmil and Eli 2014). Clinical reports indicate that parents of children with disabilities may be less likely to challenge food refusal if they believe that it is expected that their child be ‘picky’ (Kalyva 2011).

#### 3.1.2 Other Motivations for IWPD to Reduce Their Weight

Another factor that can contribute to ED/DE in IWPD is the desire to improve or maintain mobility and independence (Gross et al. 2000; Thomas et al. 2019). Studies indicate that IWPD often feel their mobility—such as walking, transferring and functional movement—is easier with a lighter body weight. Both adults and youth with disabilities and ED/DE have reported increased motivation to reduce or maintain their weight to lessen the physical burden on those assisting with transfers and daily activities (Webb et al. 2009; Taleporos and McCabe 2005). Silber et al. presented a series of case studies of five young women with spina bifida who all reported that their EDs resulted from initially pursuing weight loss to enhance mobility. In another example, Webb et al. discussed a woman with CP who expressed always feeling that she was ‘in the way’ (p. 88) due to her disability, which contributed to the development of her anorexia nervosa because she felt less obtrusive as her body became smaller (Webb et al. 2009). Therefore, IWPD may be particularly sensitized to their body size and weight when requiring others to help them transfer or engage in activities of daily living, which increases the risk of developing ED/DE (Cicmil and Eli 2014).

### 3.2 Ableism Impacting the Development of ED/DE

#### 3.2.1 The Connection Between Disability and Body Image

Body image and ED/DE are often intricately intertwined (Waldman et al. 2013). In individuals without disabilities, negative views of the self, dissatisfaction with one's weight and shape, and distortions in estimating body size have been linked to the development of ED/DEs (Waldman et al. 2013). Some researchers have specified that developing an ED is dependent upon having a preoccupation or dissatisfaction with weight and shape (Polivy and Herman 2003). A study on DE, body dissatisfaction and mental health outcomes in women with physical disabilities found that greater health issues and body dissatisfaction predicted greater DE symptoms (Roosen 2017).

Research has also reported a link between having a physical disability and poorer body image (Thomas et al. 2019). Some people with mobility impairments have also been found to devalue their body (Taleporos and McCabe 2005). Research conducted on body image and disability has primarily focused on physical disfigurements and visible differences as part of a disability and mainly on adult women (Cicmil and Eli 2014). Such work has shown that individuals with visible differences frequently experience distress due to concerns about others' perceptions of their appearance, have feelings of otherness and experience considerable stigma (Rumsey et al. 2004). Further, studies have shown that people with visible physical disabilities report increased stigma and decreased body‐esteem compared with those without disabilities (Thomas et al. 2019). This, then, can increase the risk of ED/DE in IWPD given that higher body dissatisfaction is associated with DE behaviours.

#### 3.2.2 Weight Loss as a Mechanism to Ameliorate Ableism

Societal devaluation of bodies that look different in some way than the population ‘norm’ can lead to general body dissatisfaction, mental health issues and disordered eating (Roosen 2017). When disordered eating behaviours result in weight loss, IWPD often receive praise and social validation, thus reinforcing the DE behaviours (Cicmil and Eli 2014). Furthermore, studies have found that some youth with disabilities can view weight loss as a form of achievement or ‘success,’ in addition to attempting to reduce negative comments on their appearance (Cicmil and Eli 2014; Taleporos and McCabe 2005). The idea of weight loss as an achievement can also be associated with wanting to feel more ‘normal.’ For example, in the case studies of five women with spina bifida, EDs were seen as a more ‘fashionable’ (p. 459) condition than their particular disability (Silber et al. 1999). Individuals with disabilities may therefore use weight loss to counter feelings of powerlessness relating to physical differences—especially when their weight loss is praised (Cicmil and Eli 2014). This aligns with research that shows that individuals without disabilities often turn to EDs for a sense of greater control over their environment (American Psychiatric Association 2013). For the general population, a desire to achieve the ‘thin ideal’ is perpetuated, in part, through mass media messaging (Katzman et al. 2003). For individuals with disabilities, this messaging can have complex implications. Although people with disabilities make up 16% of the world's population (Statistics Canada 2022), they are noticeably underrepresented in the media. For example, one study estimated that only about 2% of actors/actresses have a physical disability (Woodburn and Kopić 2016). This underrepresentation can cause pressure to meet body standards of individuals without disabilities (Cicmil and Eli 2014; Bordo 2003). Societal messages around smaller bodies may therefore be particularly potent for youth with disabilities if they feel pressured to meet this homogenous representation of body size and shape via social comparison (Pinquart and Behle 2021) These societal messages have been shown to contribute to decreased self‐esteem in female youth and women with disabilities (Taleporos and McCabe 2005; Bordo 2003). Other research reports that youth with disabilities rarely compare themselves to their peers with disabilities but are more likely to compare themselves to those without disabilities (Pinquart and Behle 2021). Therefore, the risk of ED/DE for IWPD may be higher in an effort to challenge ableism and stigma associated with physical differences.

## 4 Clinical Implications Across the Lifespan

Clinical research in the ED/DE field is primarily focused on individuals without disabilities. Recognizing and addressing symptoms of ED/DEs are critical, as they can impact the health and wellbeing of IWPD across the lifespan (Gravestock 2000). For children and youth with disabilities, standardized weight growth charts fail to account for differing body compositions (Sharma and Kushner 2009; Güngör 2014; Salera et al. 2017), such as progressive muscle loss or those with different muscle and fat distribution. For example, some children with spina bifida may have very small legs/lower extremities but a ‘typical’ sized torso (Salera et al. 2017; Centers of Disease Control 2011). These challenges can contribute to delays in diagnosis and appropriate management of ED/DEs and ultimately impact prognosis as the problematic eating behaviours become more entrenched (Disability Community 2022; Stiles‐Shields and Holmbeck 2020).

Youth with physical disabilities are more likely to engage in frequent weight‐related conversations with healthcare roviders (HCPs) as part of routine clinic visits (Disability Community 2022; Bryon et al. 2008). In general, weight, nutrition and appetite are more closely monitored in clinical settings than with typically developing youth (Bryon et al. 2008). Therefore, HCPs and caregivers may, inadvertently, reinforce messages around weight loss, health and mobility (Silber et al. 1999; Cicmil and Eli 2014) that increase the link between weight and self‐evaluation (Disability Community 2022; Bryon et al. 2008) and encourage DE behaviours. Additional training is needed for HCPs who work with youth with physical disabilities and their families around using appropriate, sensitive and nonstigmatizing language (Barlow and Expert Committee 2007). This is particularly important in paediatric settings (Gravestock 2000; Disability Community 2022), given that the onset of ED/DE is typically around adolescence. Unfortunately, best practice guidelines and other resources are currently lacking (Carcone et al. 2016).

Multidisciplinary care and collaboration should be mobilised to support ED/DE in all IWPD given their complex needs (Gravestock 2000). This may include physical health care and rehabilitation support, mental health care, peer support groups and social care services (Gravestock 2000). This multidisciplinary approach is particularly important given that the research reviewed in this narrative review has shown that ED/DE can stem from physical characteristics related to a person's disability, psychological challenges associated with body dissatisfaction and low self‐esteem, functional factors such as incontinence or mobility, as well as navigating ableism.

Caregivers have reported receiving insufficient and inappropriate support on how to support EDs/DE with their children with disabilities (Cicmil and Eli 2014). This is concerning given the importance of family support in the treatment of ED/DE (Roles 2005) and that family systems may be more involved than with youth without disabilities (Dillenburger and Mckerr 2014; Walker et al. 2019). Existing ED treatment programs often require patients and their caregivers attend the program for several hours a day or to stay overnight (American Academy of Pediatrics 2010). Such programs may interfere with other important health management tasks and appointments (Brown et al. 2015; Irby et al. 2012). It is therefore important for HCPs offering ED/DE treatments to understand the importance of flexibility with their patients' schedules. One way of mitigating this could be through virtual programming options, where patients may log on in the comfort of their home and participate in family, group and/or individual therapy. This has become increasingly more popular as an outcome of the COVID‐19 pandemic (Nicholas et al. 2022). HCPs could also find ways to integrate ED/DE treatment approaches within other clinic appointments.

## 5 Future Directions

There is a critical need for future research to advance our understanding of the prevalence, presentation and treatment of ED/DE in IWPD across the life span (Disability Community 2022; Stiles‐Shields and Holmbeck 2020). Future work should also aim to understand the perspectives of boys/males and other gender identities with physical disabilities, as the vast majority of the work currently focuses on females. It would also prove beneficial for future research to examine risk factors, prevalence and presentation of ED/DE in specific physical disability subgroups (i.e., spina bifida, Duchenne muscular dystrophy, spinal cord injury) to understand characteristics specific and unique to those individuals.

Lastly, it would behove future researchers to adopt an intersectional lens to better understand how overlapping identities (e.g., gender, race, sexual orientation) and systems of oppression (e.g., patriarchy, ableism) can influence the experiences of individuals with disabilities and ED/DE. This approach to research is critical in identifying more equitable and inclusive approaches to ED/DE treatment and prevention in this population.

### 5.1 Strengths and Limitations

When interpreting the results, it is important to consider the limitations, both of the studies included in this review, as well as the narrative review methodology itself. The aim of this paper was to explore the current state of the literature on ED/DE in IWPD. A narrative review was selected because of the heterogeneity of the available evidence and the lack of an existing knowledge synthesis (Baumeister and Leary 1997). There were no studies differentiating between EDs and DEs relating specifically to IWPD. While the terms are distinct (Pereira and Alvarenga 2007), we have used them both to capture as much relevant literature as possible.

The quality and amount of evidence were insufficient to conduct a different type of synthesis, such as a systematic review. Employing narrative review methodology enabled us to link together studies on different topics (e.g., ableism, eating behaviours and body image) to enhance our understanding in this complex area (Siddaway et al. 2019). Furthermore, it allowed the team to interpret the evidence using their specific expertise and interests, within a more subjectivist paradigm compared with other types of review (Sukhera 2022). Narrative review methodology may miss literature that could have been identified via a scoping or systematic review (Sukhera 2022). The limitation of including only literature published in English may have limited the scope of our findings and potentially excluded relevant data. As such, our conclusions are only reflective of research conducted in the English language. The reviewed literature also lacked strengths‐based perspectives, such as disability pride and self‐advocacy, which may serve as protective factors against the development of ED/DE in individuals with disabilities. This gap highlights the need for future research to explore what factors may mitigate the risk and development of EDs/DE in this population.

It must be noted that much of the evidence we identified was at least 10–20 years old, with some studies dating even earlier, so they may not reflect contemporary knowledge and practices. However, this provides a strong rationale for new empirical work to be conducted on this topic to ensure that both clinical interventions and research knowledge are up to date and reflect the latest standards in care.

## 6 Conclusions

Individuals with disabilities and ED/DE face unique challenges concerning body image issues, feeding challenges, mobility concerns, social stigma and ableism. These intersecting challenges may increase their vulnerability to ED/DE. It is important for HCPs to recognize these unique risk factors and, potentially, the distinctive presentation of ED/DE in IWPD, and the implications that they can have on their physical and psychosocial health. Future research is required to develop our understanding of this complex phenomenon further.

## Conflicts of Interest

The authors declare no conflicts of interest.

## Ethical Approval Statement

Not applicable.

## Data Availability

Data will not be publicly available.
